# Transcriptomic insights into the low-salinity tolerance of the sea louse *Caligus elongatus*

**DOI:** 10.1007/s00360-025-01606-3

**Published:** 2025-02-28

**Authors:** Andreas Borchel, Frank Nilsen

**Affiliations:** Sea Lice Research Centre, Department of Biological Sciences, University of Bergen, Pb. 7803, NO-5020 Bergen, Norway

**Keywords:** Betaine, Hyposalinity, Parasite, Proline, Transcriptomics

## Abstract

*Caligus elongatus* is a marine copepod ectoparasite on a wide variety of fish species. It has also been observed on fish farms cultivating Atlantic salmon and reports shows that this parasite can be a problem for the industry and for the fish’s welfare. Freshwater is used as one of the non-medical treatment methods against the salmon louse (*Lepeophtheirus salmonis*). However, the efficacy of freshwater treatment against *C. elongatus* is still unknown. This study aims to fill this gap by examining the salinity tolerance limits of both adult and copepodid life stages of *C. elongatus*. Our findings reveal that detached adult *C. elongatus* exhibit low tolerance to reduced salinity, with mortality occurring within hours at salinities below 20 ppt. In contrast, copepodid stages demonstrated a slightly higher tolerance, surviving at salinities as low as 15 ppt for one day. Adult lice attached to a host quickly detached from the fish as soon as the salinity was lower than 20 ppt, suggesting that freshwater delousing might be effective in this species. To further understand the genetic basis of acclimation to reduced salinities, we performed RNA-sequencing to assemble the first transcriptome of this species and identify differentially expressed genes. Several genes regulated upon low-salinity transfer were identified. These include genes involved in proline metabolism, energy metabolism, and the transport of various ions and betaine, an osmolyte. The potential roles of these genes in salinity acclimation are discussed within an evolutionary context, providing valuable insights into the survival mechanisms of *C. elongatus* under low-salinity conditions.

**Supplementary Information:**

The online version contains supplementary material available at 10.1007/s00360-025-01606-3.

## Introduction

The survival and well-being of aquatic animals depend strongly on water parameters like temperature, presence or absence of toxins and also salinity, which refers to the amount of dissolved salts in the water. Throughout the course of evolution a diversity of aquatic species has developed different strategies to adapt to fluctuating salinities (Rivera-Ingraham and Lignot 2017). Certain animals, termed stenohaline, can only survive within a very specific salinity range. In contrast, euryhaline organisms thrive at a wider range of salinities. Additionally, organisms can be classified based on their osmotic adaptations: Osmoconformers maintain an internal osmolarity (in their blood or hemolymph) that mirrors the external environment (water), while osmoregulators actively adjust their internal osmolarity to a level that is differing from the external environment, which is a highly energy-demanding process.

The focus of this study is the marine fish louse *Caligus elongatus* Nordmann, 1832, an organism with little knowledge about its tolerance to low salinities. *C. elongatus*, a parasitic arthropod, infests over 80 different fish species (Kabata 1979), and is most abundant in the North Atlantic (Hemmingsen et al. 2020). It belongs to the class Copepoda, the order Siphonostomatoida and the family Caligidae. Hence, it belongs to the same family as the salmon louse (*Lepeophtheirus salmonis*), although their last common ancestor is dated back more than 100 million years ago (Eyun 2017). Salmon lice are very common on both wild and farmed Atlantic salmon and infestations and subsequent treatments compromise animal welfare, cause environmental problems and give rise to economic losses (reviewed in Torrissen et al. 2013). So far, the damage caused by *C. elongatus* is much smaller, but in some areas in northern Norway, the problems related to this parasite have increased (Sommerset et al. 2023) and required treatment of the fish in a few cases. One treatment for lice-infested fish involves freshwater bathing, where salmon are exposed to freshwater for several hours on a well boat to reduce lice numbers. Various studies have shown that adult salmon lice (*L. salmonis*) are quite tolerant toward low salinities for limited timeframes, where lice attached to a fish can survive in freshwater for more than a week (Finstad et al. 1995; Hahnenkamp and Fyhn 1985), while detached lice perish within a day. Interestingly, detached lice can survive for a day in water with a relatively low salinity of 5 ppt (Andrews and Horsberg 2019). Earlier life stages display less tolerance to low salinities, with the nauplii being the least tolerant, followed by the copepodid stage (Borchel et al. 2023b). Nevertheless, freshwater treatment of Atlantic salmon against *L. salmonis* is increasing in frequency (Sommerset et al. 2023) and considered efficient, however, the mechanical effects of this treatment (i.e. the handling of the fish) seem to significantly contribute to the delousing success (Guttu et al. 2024).

Data regarding the salinity tolerance of *C. elongatus* is limited. Observational studies in natural habitats have indicated a correlation between salinity levels and the population density of *C. elongatus* on sea trout (Heuch et al. 2002). The authors postulated a lower tolerance to declined salinities compared to *L. salmonis*. A 20-min freshwater immersion has shown to effectively remove *C. elongatus* from the red drum (Landsberg et al. 1991). Experimental data on the salinity tolerance of the copepodid stage of *C. elongatus* is sparse. Animals die immediately when exposed to water of 10 ppt, while a salinity of 15 ppt leads to lower mortality but inhibits lice development (Andersen 2006). Water with a salinity of 20 ppt was better tolerated, however, mortality rates were still higher when compared to a salinity of 25 ppt.

The aim of this work was to assess the hyposalinity tolerance of *C. elongatus* and compare it between two different life stages (the planktonic and lecithotrophic copepodid stage as well as the parasitic adult female stage). Additionally, we aimed to generate and make available a draft transcriptome to simplify molecular work with this species and to determine its molecular response on the transcriptomic level upon a reduction of salinity, to the lowest shared tolerable salinity level of both analyzed life stages.

## Methods

### Animal husbandry

The used *C. elongatus* animals descend from the laboratory strain CeSenja (originating from Senja in Northern Norway), which has been cultivated in the lab for more than 3 years at the time of the experiments. The free-living stages were cultivated in a similar way as the early stages of *L. salmonis* are, in hatching wells, with a continuous flow-through of full-strength seawater (34 ppt), as described before (Hamre et al. 2009). The parasitic stages were cultivated on lumpfish (*Cyclopterus lumpus*). This cultivation was in line with an approved application to the Norwegian animal research authority (Mattilsynet, ID 26020).

### Salinity tolerance

To assess the salinity tolerance of adult female *C. elongatus*, glass beakers containing 75 ml of water with different salinities (34.5, 27, 25, 23.5, 21, 19, 17 and 10 ppt) were prepared. The salinities were chosen based on previous observations, suggesting occurrence of hyposalinity-caused mortality at around 20 ppt. Four animals were plucked from fish, put into a hatching well, which was then transferred to one of the glass beakers. These were covered with black plastic foil to prevent the lice from climbing out of the vessels. The viability was assessed (see next section) after 1 h, 7 h, 24 h, 72 h, 4 d, 6 d, 7 d, 10 d and 11 d. Otherwise the lice were left alone, the water was not changed, and the lice were kept without a host to feed on. The experiment was performed in triplicates per salinity.

To get an even better estimate of the salinity tolerance with a higher resolution, the experiment was repeated once for five salinities between 24.5 and 20 ppt (24.5, 23.4, 22.7, 21.4, 20 ppt) and a 34 ppt control, with 10 adult females per salinity. After 24 h, the activity status was determined. Animals were quickly wiped dry on a tissue and put on RNAlater for later qPCR analysis as described in the following sections. Only animals that were showing signs of life were sampled.

The experiment to determine the salinity tolerance of copepodids was similar to that for adult animals. Five to ten copepodids were transferred into a hatching well, which was transferred into a beaker with a specific salinity (34, 25, 20, 15 and 10 ppt) the next day. The survival of the animals was assessed after one day. This experiment was performed in quadruplicates.

### Viability assessment of *C. elongatus*

The viability of *C. elongatus* was determined by observing their behavior within a hatching well. A healthy louse usually swims within the hatching well or attaches to the walls. Such animals were considered as alive and active. To evaluate if lice that were lying on the bottom of the hatching well were just resting but still active, the well was lifted out of the water and then replaced in the water. The lice floated up, and those lice that moved were considered active. Some animals that did not react to such treatment were analyzed further under a stereomicroscope. Animals that showed the smallest movements of their intestine or appendages were considered moribund, whereas lice that did not even show microscopic movements were considered dead.

### Fish delousing with low salinity

To investigate whether delousing with reduced-salinity water could be a viable treatment for fish infested with *C. elongatus*, a specific experiment was designed. In total, twelve Atlantic salmon were infested with *C. elongatus* copepodids (60 copepodids per fish), by lowering the water volume and adding a defined amount of copepodids, and housed in two 500 L tanks equipped with a SW flow-through system. These two tanks were used in two independent experiments with slight modifications in the experimental design (Trial 1 & 2).

Once the lice reached maturity (41 (trial 1) and 48 (trial 2) days after infestation; 11 and 18 days after calculated onset of adult stage), the experiment commenced. In trial 1, the seawater inflow was halted, and a pipe was introduced into the tank to deliver freshwater (FW). This pipe, perforated along its entire length, ensured the freshwater mixed evenly throughout the water column. The temperature of the FW was adjusted to match that of the seawater (8.5 °C). In trial 2, the seawater (SW) inflow was originally not halted but the pipe delivering FW was added in addition, to create a slower change in salinity. Using flowmeters, the inflow of SW and FW was balanced on similar levels. When the outlet water was reaching a new stable value, SW inflow was reduced and FW inflow increased, keeping the total flow rate at a nearly constant level. These flow adjustments were performed several times until finally SW inflow was halted and only FW was delivered into the tank.

In both trials, the salinity of the outlet water was monitored closely, particularly in the initial phase of the experiment, and less frequently at the end. A filter was placed under the outlet to capture any lice detached and transported out of the tank by the water current. This filter was checked regularly, and any lice found were immediately transferred to a hatching well filled with cold SW. The filters were observed with high frequency and when a louse was detected, it was transferred into a hatching well filled with cold SW.

The experiment was concluded when no lice had been found in the filter for a period of 30 min. At this point, the FW pipe was removed, and the SW flow was reactivated. The following day, the filter was inspected for any lice that might have detached post-experiment. The fish were then anesthetized (Finquel vet., 130 mg/l, 3 min) and examined for any remaining attached lice.

### RNA-seq

To obtain samples for RNA-Sequencing, copepodids and adult females were used. The experimental setup was similar to that described for assessing the salinity tolerance. Control animals were incubated in SW of 34 ppt, while the treated animals were incubated in BW of 23.5 ppt for 24 h. Excess water of all samples was removed, before the animals were transferred to RNAlater. As control for the adults, four SW-incubated lice were taken, from the treated group four lice that were classified as active and four that were considered moribund. For the copepodids, egg strings of two or three mothers were combined in one hatching well before randomly being split into the control and treatment groups. For each sample around 50–100 copepodids were pooled. After a night at 4 °C samples were transferred to – 20 ℃ until further use. For the adults, we noted the activity status at the end of the trial.

RNA was isolated from the samples using a combination of Trizol with chloroform and subsequent purification of the aqueous phase using Direct-zol RNA cleanup kits (Zymo Research), including on-column DNase treatments as described by the manufacturer. Mini kits were used for adults, micro kits for copepodids. RNA was quantified on a Nanodrop ND1000 and quality controlled on a Bioanalyzer. Library preparation and sequencing was performed at the Genomics Core Facility (GCF) at the University of Bergen. In short, RNA samples were quantified using Qubit. Libraries were prepared for sequencing using the “Illumina Stranded mRNA Prep, Ligation “ kit. Libraries were quantified using KAPA qPCR quantification. The average library input fragment size was estimated using Agilent TapeStation. A NovaSeq SP flowcell was loaded with 18 multiplexed samples and sequencing (paired ends, 2 × 100 bp) was performed using an Illumina NovaSeq6000 instrument. After sequencing, the data was demultiplexed and converted into FASTQ files. Read quality was analyzed using FastQC and visualized in MultiQC (Ewels et al. 2016).

### Computational analysis

The reads from 10 samples were employed in the de-novo RNA-Seq assembly. Each of the conditions described in this article (copepodid: SW, BW; adult: SW, BW active, BW moribund) was represented in the assembly as well as five samples generated in a previous experiment in our research group consisting of different *Caligus elongatus* tissues (legs, intestine, labial gland, ovary, egg). Due to computational limitations, it was impossible to include reads of all analyzed samples in the assembly; however we assume that most of the relevant transcriptome of the species (with the exception of male-specific genes) is represented, as all salinity-conditions were included. The transcriptome was assembled using Trinity (Grabherr et al. 2011) on a Galaxy web server (The Galaxy Community 2022). Assembly statistics were generated using Transrate (Smith-Unna et al. 2016). Most likely ORFs were predicted using TransDecoder (Haas et al. 2013) and the predicted peptides were blasted against protein databases containing all known copepod sequences (downloaded from protein database at NCBI), as well as the proteins encoded by the *L. salmonis* genome (Skern-Mauritzen et al. 2021). The assembly completeness was assessed using BUSCO (Manni et al. 2021). Additional blast-searches were performed against the Swissprot database, and signal peptides predicted using SignalP (Dyrløv Bendtsen et al. 2004). The gene names of the *L. salmonis* orthologues were used for a GO-term enrichment analysis of differential expressed genes using ShinyGO (Ge et al. 2020), considering GO-terms with p-values smaller than 0.1 as enriched.

For quantification of the transcripts, Salmon v.1.9.0 (Patro et al. 2017) was used in mapping-based mode employing the gcBias and validateMappings parameters, mapping the reads against the de-novo transcriptome created before. Differential expression analysis was performed using DESeq2 (Love et al. 2014) after import of the reads via tximport (Soneson et al. 2016) and pre-filtering of lowly expressed genes (< 10 mapped reads) in R (R Core Team 2020) employing R Studio (RStudio Team 2020). We compared the gene expression between active adults in SW and BW, between copepodids in SW and BW, and between active and moribund adults in BW. Each comparison was performed in a separate analysis. Fold-changes and adjusted p-values were calculated using DeSeq2’s lfcShrink function setting the “type”-parameter to”normal” (Love et al. 2014). The adjusted p-values were calculated with two different settings: Once with a set fold change threshold of 1.5 (parameter lfcThreshold = 0.585) and once without such a threshold. An additional comparison was performed to identify genes regulated by the salinity independent of the life stage of the animals. To this end, both factors (salinity and life stage) were included in the same model. NA-values obtained for adjusted p-values due to independent filtering were set to 999 to simplify sorting in the resulting files.

### qPCR

To validate RNA-Seq results, new experiments with new animals were performed. The incubation time was 24 h, the salinities tested were SW (34.5 ppt) and BW (23.5 ppt). The water temperature was 11 °C. Six animals per salinity were analyzed for the adult life stage, four (pooled) samples per salinity for the copepodids. RNA was isolated using the same methods as described for the RNA-Seq samples. Complementary DNA was synthesized using the Affinityscript qPCR cDNA synthesis kit with a blend of Oligo (dt) and random hexamer primers. 200 ng (adults) or 90 ng (copepodids) of RNA were employed in the reactions. Copepodid cDNA was diluted 1:5, adult cDNA 1:10.

Target genes to be analyzed (Table S1) were selected based on the list of DEGs obtained in the RNA-Seq experiment. We mainly selected genes encoding for enzymes, as their function is relatively well understood, and genes found to be differentially expressed in both adults and copepodids, potentially suggesting a conserved role in osmoregulation between the life stages. An additional focus was put on homologues of genes known to be regulated in low salinity in *L. salmonis* (Borchel et al. 2021)*.*

Quantitative PCR reactions were run on Quantstudio 3 qPCR machines, using PowerUp SYBR Green Master Mix. The reaction volume was 10 µl, including 2 µl of cDNA. Primer concentration was 500 nM for forward and reverse primer, respectively. Primer sequences are given in Table S1. The following thermocycling parameters were used: initiation, 50 °C for 2 min; holding, 95 °C for 2 min; 40 cycles of 95 °C for 15 s; and then 60 °C for 1 min. All samples were run in technical duplicates. Primer efficiencies were determined using relative standard-curves, with at least 5 points in two- or fivefold dilutions. Gene expression was normalized to the expression of the reference genes EIF1A and MTMR2 (Borchel et al. 2023a), which were found most stable under changing salinities as determined by a Normfinder analysis among five genes. Statistical testing for differences in gene expression was performed by paired (for copepodids) and unpaired (for adults) t-tests, performed in R employing the ggpubr package.

The samples gained from the “high-resolution” salinity tolerance experiment were used for qPCR in a similar fashion, for the control animals and the two groups in which moribund animals occurred (22.7 ppt & 21.4 ppt). Only the genes found to be differentially expressed between active and moribund animals by RNA-seq were measured. Numbers of lice analysed varied based on the available material (SW, active: n = 10; 22.7 ppt, active: n = 8; 22.7 ppt, moribund: n = 2; 21.4 ppt, active: n = 4; 21.4 ppt, moribund: n = 5). Gene expression calculation was performed as described in the previous paragraph.

## Results

### Salinity tolerance

**Fig. 1:**
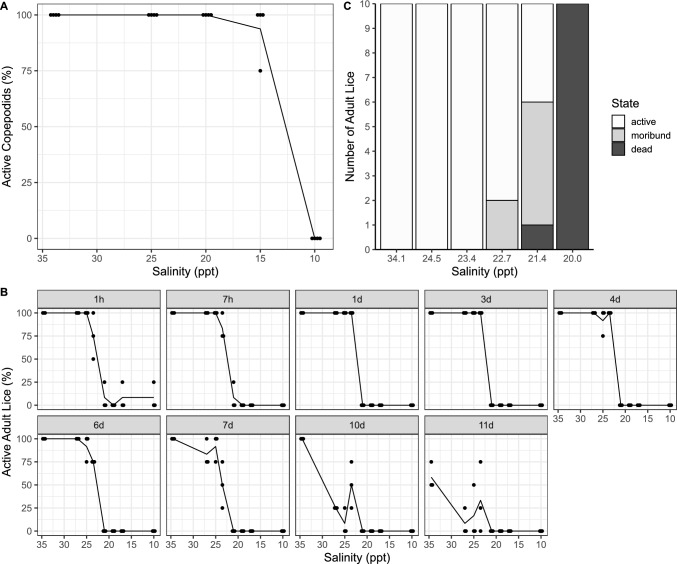
Status of *C. elongatus* in reduced salinity. **A** Visibly alive copepodids after 1 day of incubation in different salinities. Each dot represents the measurement from one replicate sample, the lines connect the means of all samples per salinity. A small jitter was added to the x-values for improved visualization. **B** Surviving adult females after 1 h–11d incubation without a host in different salinities. **C** High-resolution analysis of survival of adult females from an additional experiment in water of different salinities for 24 h. Animals were classified as active (swimming or attaching to the wall), moribund (only microscopic movements of appendages) or dead (no movements at all)

*C. elongatus* adult females and copepodids were incubated for different time spans in different salinities and their survival rate was determined (Fig. 1). The survival of copepodids was not affected by salinities down to 20 ppt. However, at 15 ppt, the survival was reduced to 75% in one of the replicate experiments, but unaffected in the other replicates, and no animals survived at 10 ppt (Fig. 1A). Despite the general survival of the lice at 15 ppt, these animals were less actively swimming in the water column and were more often located at the bottom of the hatching wells.

Adult females exhibited less tolerance towards a reduction in salinity. In salinities of 21 ppt or lower, very few active animals were observed after just one hour of incubation. At 23.5 ppt, a few inactive lice were identified after 1 h and 7 h, but these lice recovered and were active again after 24 h. By the sixth day, the number of active lice at 23.5 ppt had decreased to 75%, while almost all lice were still active at higher salinities. After 10 days, lice kept in full SW (34 ppt) were still active, while activity in all other groups had decreased to 50% or less. On the last day of the experiment (day 11), the first animals in the control SW group also became inactive (Fig. 1B).

In an additional experiment focusing on the survival of adult lice in salinities between 20 and 25 ppt with higher resolution (Fig. 1C), all animals were found active after a 24-h incubation at 23.4 ppt. However, the first moribund animals (20%) appeared when the salinity was lowered to 22.7 ppt. At 21.4 ppt, the proportion of moribund animals increased to 50%, and 10% of the animals were completely dead. At 20 ppt, all animals were found dead after the 24-h incubation.

### Delousing

**Fig. 2:**
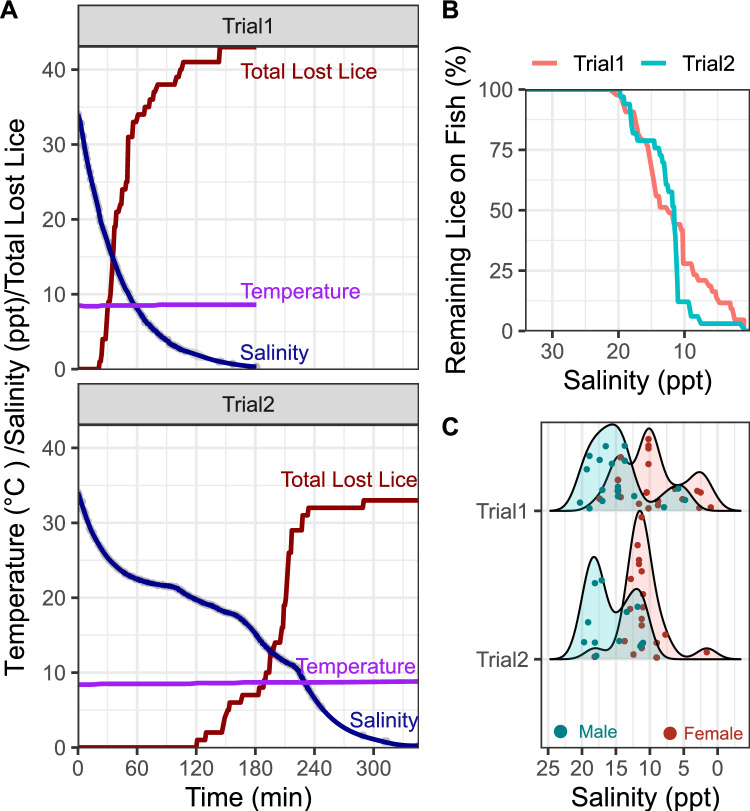
Loss of adult *C. elongatus* from Atlantic salmon during a reduction in salinity. At the beginning of the experiment, SW provision was halted, and FW delivery commenced, continuously mixing the water within the tank with new FW, reducing the salinity (Trial 1). In Trial 2 the salinity was stepwise reduced by mixing SW and FW in different ratios. Lice were collected from a filter from the outlet water of the tank as soon as they were observed. **A** The blue lines represent moving regressions of the salinity measurements. The red lines represent the number of total lost lice. The water temperature throughout the experiment is shown in purple. **B** Based on the data from A, the ratios of the remaining lice on fish were calculated based on the total number of lice for each trial, at a given salinity. **C** Sex-dependent lice loss from the fish from A. Each dot represents a lost louse

We performed two trials with *C. elongatus* attached to Atlantic salmon and observed their detachment during a salinity reduction (Fig. 2). In the first trial the salinity was reduced quicker than in the second, in which the water mixing ratios were adjusted several times (Fig. 2A). In the first trial, the first louse in the filter was found after 23 min, when the salinity had reached 20.3 ppt. Within the next 30 min, the salinity dropped to 10.2 ppt and the number of lice in the filter increased to 31. After a total of 90 min the salinity was down to 3.9 ppt and 38 lice had been found. The last lice in the filter during the FW treatment were found after 144 min, summing up to 42 lice in total, at a salinity of 1 ppt. During the next 30 min, no further lice loss was observed, and the freshwater flow was stopped, and SW flow reestablished. In a parallel tank, which was running on SW the whole time, no lice loss was observed during this period. The next day, three dead lice were found in the filter, and one louse was found on a fish, still attached, but dead. Examination of the lice that were collected from the filters and placed in hatching wells with SW revealed that all lice collected within the first 45 min of the experiment (salinity down to 11.6 ppt), recovered in SW and were still alive. Afterwards, most lice did not recover and were dead, however there were still two lice, that had survived a salinity down to 2.6 ppt. Interestingly, hatching of egg strings was observed in several hatching wells housing the lice collected in this experiment after some days, including one louse that had been on the fish for 107 min, experiencing a salinity down to 2.6 ppt. In the second trial, lice loss began after 2 h, when the salinity reached 20 ppt. During these two hours, and two hours before the start of the experiment, no lice loss was observed. Overall, the patterns in trial 2 and trial 1 were rather similar (Fig. 2B). In both cases 50% of the lice have been lost at around 12 ppt. However, the subsequent drop in lice numbers was then sharper in the second trial compared with the first trial, where the remaining decline was more gradually. Also in the second trial, most detached lice survived and recovered in SW, and nauplii hatched from several egg strings. Interestingly, in both trials sex-specific differences in the detachment pattern of the lice were observed with males beginning to detach first, at higher salinities, and females on average detaching later, at lower salinities (Fig. 2C).

### RNA sequencing & transcriptome assembly

Overall, RNA from 18 samples was sequenced, with read numbers between 36.5 and 61.9 million reads and an average of 49 million reads (Table 1). Raw reads are deposited in the Short Reads Archive with BioProject number PRJNA922488. The reads had overall a high quality and did not contain any adapter sequences or overrepresented sequences (File S2).

**Table 1:** RNA-seq libraries

Stage	Status	Water	Average read number (× 10^6^) ± SD (n)
Adult	Active	BW (23.5 ppt)	44.4 ± 4.4 (4)
Adult	Moribund	BW (23.5 ppt)	52.2 ± 6.9 (4)
Adult	Active	SW (34.1 ppt)	50.3 ± 5.9 (4)
Copepodid	Active	BW (23.5 ppt)	48.4 ± 10.3 (3)
Copepodid	Active	SW (34.1 ppt)	47.9 ± 3.3 (3)

A selection of the libraries and previous data (see methods) was used to generate a de-novo transcriptome. This consisted of 182,593 isoforms belonging to 95,021 genes. Considering only the longest isoform per gene, the average contig length was 636 bases. N50 and N90 values of all the contigs were 1793 and 313 bps, respectively and 1139 and 240 bps, when only considering the longest isoform per gene. Transrate identified high confidence predicted homologs for 16% of the contigs when using a *L. salmonis* protein database as reference, whereas 73% of these reference proteins were assigned a homolog in the *C. elongatus* transcriptome assembly. Running Busco to analyze the completeness of the transcriptome assembly, yielded the following results when running against arthropoda_odb10 (Creation date: 2020–09–10, number of genomes: 90, number of BUSCOs: 1013) in transcriptome mode: 96% complete BUSCOs, with 40.7% single-copy and 55.3% duplicated BUSCOs, 1.3% fragmented BUSCOs and 2.7% missing BUSCOs. The transcriptome is deposited in the Transcriptome Shotgun Assembly (TSA) database with accession GKUH00000000, and can also be found in Supplementary file S3, with its annotation in supplementary file S4.

### Differentially expressed genes

Both copepodids and adults were subjected to a 24-h exposure to either BW of 23.5 ppt or full-strength SW. This salinity was chosen as the lowest salinity bearable for both life stages (for 24 h), as determined in the salinity tolerance experiments. Following this exposure, samples were collected for gene expression analysis. The adults that were exposed to BW were further categorized into active and moribund groups, based on their observed behavior at the time of sampling. All the results from differential expression analysis are given in Supplementary table S5. Mapping rates for all libraries were between 97 and 98%.

**Fig. 3:**
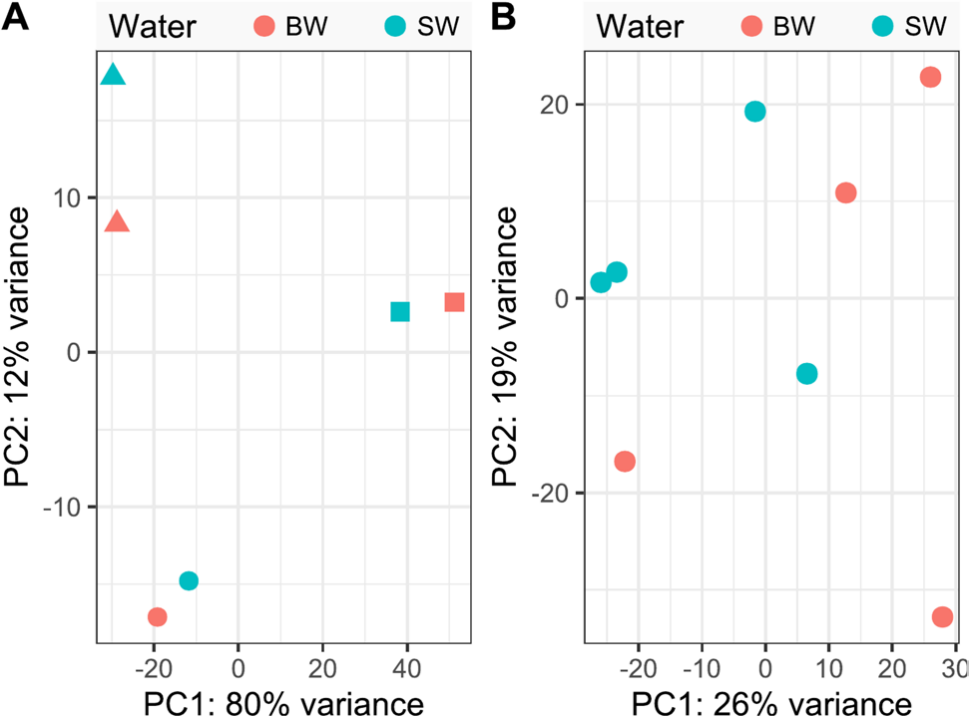
Principial component analyses of the RNA-Seq data. **A** Copepodid analysis. Colors encode the salinity (sea water, SW, 34 ppt and brackish water, BW, 23.5 ppt), same symbol shapes indicate that the samples origin from the same egg string pool. **B** Adult female analysis, considering salinity as factor, only including active lice

To assess the overall similarity and possible clustering of the samples, we conducted principal component analyses. The samples showed minimal separation based on the salinity treatment (Fig. 3). In the case of copepodids, samples originating from the same egg string pool clustered closer together than samples exposed to the same salinity. For adults, no distinct separation was observed between BW and SW samples.

**Fig. 4:**
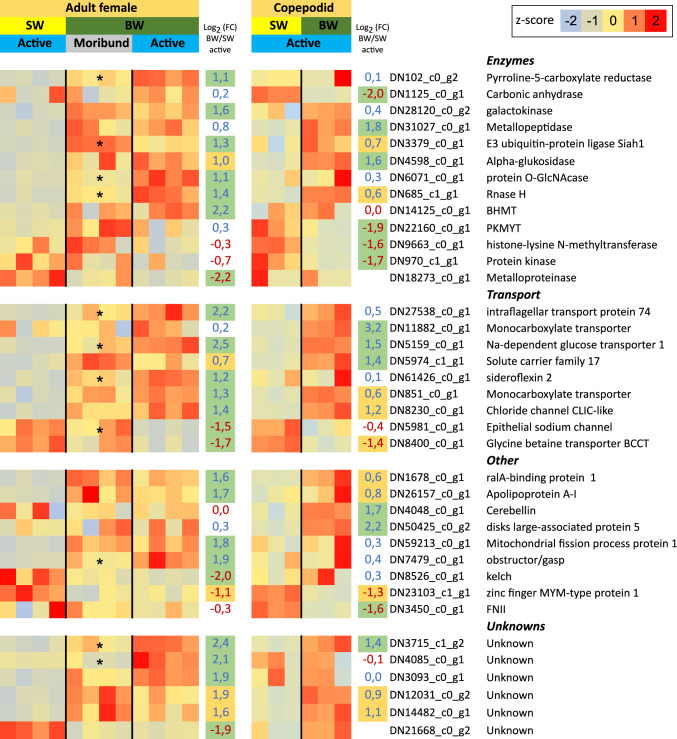
Gene expression of differentially expressed genes (DEGs) in *C. elongatus* copepodids and adult females in SW and BW (23.5 ppt). Each square represents the row z-normalized expression value, with red representing high expression and blue low expression. Normalization was performed for adult females and copepodids separately. The Log_2_ (FC)-values are written in blue for upregulated genes and red for downregulated genes. FCs with green background were significantly different from an FC of 1.5, an orange background indicates FCs which were found to be significantly different from 0. Missing FCs are due to the filtering of low expressed genes. Several other DEGs for which no CDS could be determined were omitted from this graph. Asterisks (*) mark significant differences between moribund and active BW-incubated females

The number of genes differentially expressed in response to salinity was relatively small. In copepodids, 16 genes showed higher expression following exposure to BW (FC ≥ 1.5, p_adj1.5_ ≤ 0.1), and 6 genes showed lower expression, resulting in a total of 22 DEGs. In the active adult group, 26 genes were stronger expressed after BW treatment, while 10 genes were lower expressed (Fig. 4).

Comparing the DEGs from copepodids and adults exposed to BW, only two genes were shared by both life stages. These genes encode a novel, unknown protein and a sodium-dependent glucose transporter, respectively. Notably, both genes were upregulated in BW. However, several of the genes found significantly different expressed for a |FC| of at least 1.5 in one life stage, also were significantly different from zero in the other life stage.

When integrating both water type and life stage into one model for the analysis, a higher number of DEGs was obtained for the comparison between SW and BW. Specifically, three genes were expressed stronger in SW (p_adj1.5_ ≤ 0.1), while 18 genes were more strongly expressed in BW. Among these, only two genes were annotated: the previously mentioned upregulated sodium-dependent glucose transporter and a downregulated glycine betaine transporter.

Interestingly, some of the genes regulated by active adult lice in BW showed significant differential expression between active and moribund animals. Of a total of fifteen such genes, eleven were expressed at lower levels in moribund animals compared to active ones, while four genes were stronger expressed in moribund animals.

Overall, the salinity-related DEGs included several enzymes, membrane transporters and various other proteins, besides several genes without annotation (Fig. 4). As only a few genes were found differentially expressed between the salinities with the presented filter settings, a GO-term enrichment analysis was not meaningful for these genes. Therefore, this analysis was performed for genes that were found differentially expressed without a FC-threshold (|FC|> 1, p_adj_ ≤ 0.05). For the genes upregulated in copepodids in BW an enrichment of genes involved in “DNA metabolic process” (p = 7E-5), “cellular response to DNA damage stimulus” (p = 0.03) and “cellular response to stress” (p = 0.07) was found. For the downregulated genes, an enrichment was found for “transmembrane transport” (p = 0.002) and “transporter activity” (p = 0.05). For upregulated genes in adults, the highest enrichment was found for “proline metabolic process” (P = 0.08), “glycolytic process” (p = 0.06) and “ATP generation from ADP” (p = 0.06). No gene enrichment was detected for the downregulated genes in adult females.

### Verification via qPCR

**Fig. 5:**
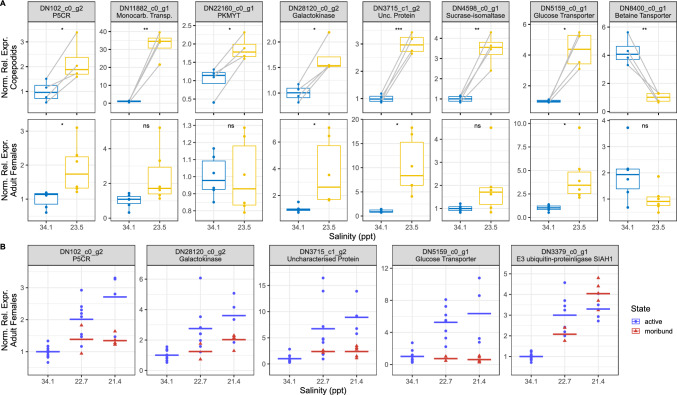
Gene expression as measured by qPCR. **A** Expression of selected genes in copepodids (upper row) and adult females (lower row) after exposure to SW (34.1 ppt, blue) or BW (23.5 ppt, yellow) for 1 day. For the copepodids values from animals originating from the same egg string are connected with gray lines. Significance levels from paired (copepodids) or unpaired t-tests are shown by asterisks. ns = not significant. **B** Expression of selected genes after incubation of adult lice in different salinities (same lice as those in Fig. 1 C), separated according to the animals’ status (active, moribund) after 24 h incubation. *P5CR* pyrroline-5-carboxylate reductase, *Monocar*. *Transp*. monocarboxylate transporter, *PKMYT* membrane-associated tyrosine- and threonine-specific cdc2-inhibitory kinase, *Unc*. *Protein* Uncharacterized protein

To validate the findings of the initial RNA-Seq experiment, we conducted a new experiment using new, independent samples from animals in both the adult and copepodid stages. These animals were incubated in water with a salinity of 23.5 ppt for a duration of 24 h. The samples obtained were then subjected to qPCR analysis, focusing on a selection of eight genes with differential expression in at least one of the stages after BW treatment in the initial RNA-Seq experiment. Surprisingly, this time, none of the animals were found to be moribund at this salinity, in contrast to the animals from the RNA-Seq experiment. At the gene expression level, the findings from the RNA-Seq experiment were generally confirmed also by this experiment (Fig. 5). For the adult lice, five of the analyzed genes were found to be significantly regulated in the RNA-Seq-experiment (P5CR, galactokinase, glucose transporter, glycine betaine transporter, unknown), and four of these were regulated when analyzed by qPCR, with the exception for the glycine betaine transporter. However, there was a trend towards a downregulation of this gene in the qPCR data also, similar to the observation in the RNA-Seq experiment. All the eight analyzed genes were significantly differentially expressed in copepodids incubated in SW and BW. In the RNA-Seq data four of these were significantly different with a FC of at least 1.5, and six of them without a defined minimum FC. The galactokinase and the pyrroline-5-carboxylate reductase were not significantly regulated in the RNA-Seq data. Only one gene (PKMYT) had opposing regulation in the RNA-Seq and the qPCR data.

To validate the findings from RNA-Seq that several genes were differently regulated between lice that were actively swimming in the water with low salinity and lice that appeared moribund, samples from the high-resolution experiment (see Fig. 1C) were analyzed using qPCR for salinities of 34.1 ppt, 22.7 pt and 21.4 ppt. Overall, the RNA-Seq results were confirmed in these new samples. For all five analyzed genes, their expression increased in active animals in 22.7 ppt as well as 21.4 ppt compared to the SW controls. However, the upregulation was lower in moribund animals compared to active animals in four out of five cases. While there were indications of a lower upregulation for most genes in moribund animals, the glucose transporter gene was unregulated in moribund animals, but strongly upregulated in active animals. On the other hand, the E3 ubiquitin-proteinligase SIAH1 was found to be equally upregulated at 21.4 ppt in active and moribund animals with a trend for higher upregulation in the moribund animals, similar to the RNA-Seq results.

## Discussion

### Salinity tolerance

The copepodids examined in this study had a higher tolerance for reduced salinities than the *C. elongatus* copepodids in the only other published study on this topic. Andersen (2006) found survival rates of only 23–37% for animals incubated for 24 h in 15 ppt. In this present study, the survival rate at the same salinity was between 75 and 100%. However, it was apparent that the lice incubated in this salinity were less active and more on the bottom than swimming in the whole water column, suggesting that this low salinity was still detrimental for the copepodids. At 20 ppt all animals in the current study survived, whereas Andersen (2006) found mortality rates between 16 and 26%. The exact reasons for the discrepancy between these findings remain unclear but it might be due to technical differences and/or differences between the lice-strains used in the two studies. The lice used in the present study were from the lab strain CeSenja, while the lice used by Andersen (2006) were collected from wild lumpfish in 10 m depth at Norway’s east coast and eggs were hatched in the lab. The lab strain CeSenja has only been exposed to full-strength SW for several generations, whereas the history of the wild lice is unknown. Nevertheless, both studies show some similar trends. A salinity of 10 ppt seems to be close to the lower salinity limit for survival of *C. elongatus* copepodids and they might even die instantly when facing this salinity (Andersen 2006). At 15 ppt survival is general possible for a certain timeframe, and at least one animal was found to survive for 5.5 days in this salinity, while at 20 ppt even longer survival times were observed (Andersen 2006). Despite survival at 23.5 ppt, genes involved in “cellular response to DNA damage stimulus” and “cellular response to stress”, were upregulated at this salinity, suggesting that these genes might facilitate survival in these apparently suboptimal conditions.

For the salmon louse (*L. salmonis*), the tolerance of the copepodid stage to reduced salinity varies between different studies. In some studies rather low tolerance to low salinities was evident with less than a day of survival in 26 ppt (Bricknell et al. 2006; Sievers et al. 2019), whereas Andrews and Horsberg (2019) found that some lice strains survived for 24 h in water with 19 ppt. Hence, *C. elongatus* copepodids seem to tolerate similar salinities as *L. salmonis* copepodids.

Our results show that the tolerance of *C. elongatus* varies significantly between different life stages (copepodid and adult). An incubation of 24 h in 20 ppt led to the death of all adult lice whereas all copepodids survived in this salinity and tolerated an even lower salinity of 15 ppt. Interestingly, this ratio is the opposite from that observed in the salmon louse (*L. salmonis)*, where copepodids have a lower tolerance for low salinities than adult animals (Andrews and Horsberg 2019). The differences for the adults are especially striking, as adult female *L. salmonis*, detached from a host, can survive a 24- hour exposure in as low as 5 ppt (Andrews and Horsberg 2019), whereas *C. elongatus* not even survives an isochronal incubation at 20 ppt. This can be the explanation why freshwater delousing had an even higher effect on *C. elongatus* than *L. salmonis* (Guttu et al. 2024). Reasons for this difference are so far speculative. On the functional side, there is an obvious size difference between both species, with *C. elongatus* females being almost half the size of *L. salmonis* females (Johnson and Albright 1991). One might anticipate that larger animals, due to their higher surface area, would be more exposed to the external environment, potentially making them more sensitive to environmental changes in salinity. Conversely, it has been shown that both sexes of *L. salmonis* can tolerate 5 ppt (Andrews and Horsberg 2019) in spite of the males being significantly smaller than the females. Therefore, the size differences seem not to be the decisive factor. It has been suggested that adult *C. elongatus* are better swimmers than *L. salmonis* and could thereby have chances to leave and swim away from a host which is staying in areas with lower salinity (Andersen 2006). As *C. elongatus* has many more host species than *L. salmonis* it might be less risky for this species to leave its host and try to infect a new host and be able to stay at the preferred salinity, thereby potentially reducing the necessity for low-salinity tolerance.

For the adults, we observed some animals reaching a moribund state at which they would still move their appendages but did not actively swim. The threshold for this status varied slightly in the different experiments. In the RNA-Seq experiment several animals were moribund at 23.5 ppt, while none were in the repeated experiment for sampling the qPCR samples. The “high resolution” experiment then found 50% of the animals moribund at 21.4 ppt. This suggests that either the salinity measurement was not perfectly reliable or that there is a certain variation between the lice regarding what kind of salinity they can endure, before reaching the moribund state. Genetic factors or other factors like age and nutritional state might contribute.

A closer relative to *C. elongatus* than *L. salmonis* is *Caligus rogercresseyi*, the most common ectoparasite in Chilean salmon aquaculture. For this species more than 50% survival of adult lice was observed after an incubation in 20 ppt for 24 h (Bravo et al. 2008) and some animals even survived for one day in 5 ppt. Overall, for the three sea louse species with available salinity tolerance data from their adult stage, *L. salmonis* is most resistant to a reduction in salinity, followed by *C. rogercresseyi*, and *C. elongatus* is most susceptible to reduced salinities with a rather narrow tolerance window for normal survival.

The delousing experiment shows that being attached to a fish does not prevent the detrimental effects of low salinity on *C. elongatus*. After three hours in a gradually reduced salinity from 34 to 1 ppt (trial 1), no living lice were found on the fish. Already after 45 min, when the salinity had reached 11.6 ppt, half of the lice were detached from the fish. The recovery of most of the detached lice in SW indicates that the lice were rather stunned than dead. It is unclear if the lice are unable to uphold the attachment to the fish in low salinity or if it they actively leave the host as a survival strategy and search for a new host at a more suitable salinity. The second trial with a slower reduction in salinity, obtained quite similar results as the first trial with 50% of the lice detaching at around 12 ppt. However, differences in the speed of detachment indicate that the rate of salinity drop might also play a role in the effectiveness of the treatment. For aquaculture, these findings are good news, and freshwater delousing as used for *L. salmonis* can be used against *C. elongatus* with most likely higher efficacy. The results suggest that for example as little as 2 h at 10 ppt might be enough to clear a fish of adult *C. elongatus* without handling of the fish. For commercial delousing operations it is important that filters are in place to remove detached lice from the outlet water, to prevent surviving sea lice from reattaching to the fish after treatment. Also egg strings must be prevented to be flushed out into the ocean, as the embryos apparently survive within the egg string for some hours in low salinity, similar to salmon louse egg strings (Borchel et al. 2023b).

### A transcriptome for *C. elongatus*

So far, very little genetic information is available for the *C. elongatus*. To the date (September 2024), only 41 sequences have been deposited in GenBank. No ESTs, transcriptome or genome are available. Therefore, to be able to identify gene regulation, a de-novo- transcriptome assembly based on RNA-Seq data was generated. This can be challenging, however, a high percentage of complete BUSCOs (96%) was obtained, suggesting that the created transcriptome has a high quality and contains most of the evolutionary conserved genes expected in the arthropod lineage. Many of the BUSCOs were found to be duplicated, which is likely caused by the many isoforms of the genes created by the Trinity assembler. But even when considering only the genes that were assembled, their number was remarkably high (95,021). In comparison 18,525 genes were identified in the genome of *C. rogercresseyi* (Núñez-Acuña et al. 2023), while an earlier de-novo transcriptome had yielded more than 80,000 contigs with 24,000 annotated genes (Gallardo-Escárate et al. 2014). For *L. salmonis,* gene numbers based on the genome were 13,081 (Skern-Mauritzen et al. 2021) or 19,181 (Joshi et al. 2022). De-novo transcriptomes created from data from long-read sequencing found 31,092 transcripts originating from 10,034 genomic loci (Hansen et al. 2023). This suggests that not all assembled transcripts actually represent a correct gene. Besides technical problems due to the library generation or during the assembly, which might lead to fragmented genes counted as several genes, a certain amount of these transcripts is probably not protein coding. Several of the assembled genes apparently also had a low read support. For example in the DEG analysis of the adults, only 23,000 genes were actually considered after pre-filtering and independent filtering. Therefore, these potential incorrect contigs do not pose a problem for the DEG analysis. However, a future transcriptome encompassing long reads or even better a genome of this species will allow for better annotation and thereby understanding of gene regulation in this species.

In the case of copepodids, the differences between the animals from different egg strings were more pronounced than the differences caused by the low-salinity treatment. The precise age composition of the various samples has not been recorded. Therefore, it is not possible to deduce if these differences are due to genetic or epigenetic differences, or different developmental times of the animals. Instar age is known to influence gene expression in *L. salmonis* (Eichner et al. 2015). For instance, molting-related genes are strongly induced briefly before molting. Moreover, unequal sex ratios in the samples could theoretically contribute to the observed gene expression differences. From the nauplius I stage onwards, male *L. salmonis* develop faster than their female counterparts and vary in gene expression (Borchel et al. 2022). Nevertheless, for the analysis of gene expression differences due to salinity reduction, the egg string pools which the copepodids originated from were included as a factor for the calculations. The copepodids from two to three egg strings were first pooled and then split into control and treatment group, so that they were paired samples, with a similar mix of lice. Significant differences found due to low salinity despite the differences between the different egg string pools are thereby the conserved copepodids response, happening independently of other factors like age and composition, and might thereby be most relevant.

### Transcriptomic acclimation to low salinity

Rather few genes were found differentially expressed after BW (23.5 ppt) treatment. However, the used filter criteria were rather strict, so that the resulting DEGs are estimated rather conservative. The number of DEGs identified in BW in *L. salmonis* (with the same filtering criteria) was not much higher, although the BW used there was less saline with 12–16 ppt (Borchel et al. 2021). In this study, water with a salinity of 23.5 ppt was used for adults and copepodids. While the salinity was the lowest tolerable salinity for the adults, the copepodids could have been exposed to even lower salinities without inducing mortality. A future study might analyze the gene expression of copepodids to lower, almost-deadly salinities to identify further genes involved in the survival of low salinities.

To validate the RNA-Seq-based results, the experiment was replicated with new samples and selected genes were measured by qPCR. The qPCR results confirmed the RNA-Seq results very well (with the exception of PKMYT), suggesting the validity of the DEG analysis. Some of the genes (i.e. those that were analyzed in the moribund animals as well) have even been found to be regulated in a total of three independent experiments, demonstrating a high validity of the results.

### Proline metabolism

A pyrroline-5-carboxylate reductase (P5CR) was upregulated in adult *C. elongatus* exposed to BW in all experiments. Additionally, this gene was also upregulated in copepodids exposed to BW based on the qPCR analysis, but not by the RNA-Seq data.

The transcriptome contained a total of three genes that were annotated as P5CR based on the blast results of the encoded peptides, but only one of these was significantly regulated by the water treatment. P5CR is one of two enzymes involved in the biosynthesis of proline; the other is delta-1-pyrroline-5-carboxylate synthase (P5CS). Although P5CS was found in the transcriptome with one copy, it was unaffected by salinity. In plants, in which proline is an important osmolyte under salinity stress (El Moukhtari et al. 2020), P5CS is the rate limiting step of proline biosynthesis (Liang et al. 2013).

Upon applying less stringent filtering conditions, we found that proline dehydrogenase, an enzyme involved in proline catabolism and catalyzing the reaction opposite to P5CR (Servet et al. 2012), was upregulated (1.7-fold) in adults under BW conditions.

The upregulation of one of the two enzymes involved in proline synthesis under BW conditions is particularly intriguing since similar regulation has been observed in other copepods. Both the salmon louse *L. salmonis* (Borchel et al. 2021) and the harpacticoid copepod *Tigriopus californicus* (DeBiasse et al. 2018) increase their gene expression of P5CS and P5CR upon transfer from SW to low salinity water. Thereby, a total three copepod species have so far been identified, which modify the gene expression of enzymes involved in proline metabolism, after exposure to a reduced salinity. This suggests that this might represent an evolutionary conserved osmoregulatory adaptation for copepods and more species (both parasites and free-living) should be analyzed to see if this is a general mechanism in copepods. In addition, further studies at the proteomic and metabolic levels should be performed to fully understand the role of proline metabolism under low environmental salinity conditions in these species.

### Energy metabolism

Several genes encoding for proteins involved in the energy metabolism were regulated by changes in salinity. One of the upregulated genes was galactokinase. This gene is involved in the Leloir pathway which converts galactose to glucose-1-phosphate (Thoden and Holden 2003), which can further be transformed to glucose. Additionally, a sucrase-isomaltase, or according to another annotation, an alpha-glucosidase was found to be upregulated in BW-exposed copepodids. This enzyme is involved in the breakdown of complex sugars into glucose-monomers (Krasikov et al. 2001). Finally, a sodium-dependent glucose transporter was upregulated in adults and copepodids in BW in the samples analyzed by RNA-Seq and the samples analyzed by qPCR. Interestingly, a sodium/glucose cotransporter was upregulated in the gills of the crab *Carcinus maenas* upon exposure to reduced salinity (Towle et al. 2011). In adult female salmon lice, on the other hand, the same gene was downregulated in attached lice after 24 h in freshwater (Borchel et al. 2021). Altogether, several genes involved in the provision of glucose are regulated by low salinity. This can contribute to necessary energy for osmoregulation, which is known as an energy-demanding process in marine invertebrates (Rivera-Ingraham and Lignot 2017). Additionally, changes in glucose metabolism can be due to the involvement of glucose in the stress response. Hyperglycemia as response to environmental stressors is known in Crustaceans (Lorenzon 2005).

### Glycine betaine transporter

A protein annotated as glycine betaine transporter (BCCT transporter) was downregulated in *C. elongatus* copepodids and adults when exposed to low salinities (23.5 ppt). Glycine betaine is an important osmolyte in bacteria, plants, mammalian kidneys and several marine invertebrates (Fedotova and Kruchinin 2017). BCCT transporters are widespread within the domain of bacteria, in eukaryotes only few groups are known to have these genes, among them Cnidaria like corals, dinoflagellates and cryptohytes (Ngugi et al. 2020). Based on data from Ensembl Metazoa, there are also BCCT homologues within the copepods *L. salmonis*, *Eurytemora affinis* and *Tigriopus californicus*. Despite having the transporter, *L. salmonis* seems to lack the capability to produce glycine betaine itself as its genome does not encode a betaine aldehyde dehydrogenase. In our *C. elongatus* transcriptome we were unable to identify such an enzyme as well. Changes in the concentration of betaine in dependence of salinity were found in other crustaceans like the shrimp *Litopenaeus vannamei* (Delgado-Gaytán et al. 2020) and the copepod *Apocyclops royi* (Winding Hansen et al. 2022). In microbes most of the BCCT transporters are related to the adjustment to different osmolarities and several of them are regulated on the transcriptional level, i.e. being induced in high osmolarity environments (Ziegler et al. 2010). Also in *L. salmonis* males and females a downregulation of a BCCT transporter was observed in BW, but less so in freshwater (Borchel et al. 2021). Glycine betaine can be detected in SW (Beale and Airs 2016). Potentially, *C. elongatus* takes it up from the sea via the BCCT transporter, as it lacks the capability to synthesize it itself. In water with reduced salinity, there might be a smaller need for import of this substance, thereby leading to a downregulation of the transporter. Nevertheless, a more detailed analysis of the putative BCCT transporters in copepods is crucial. The long evolutionary distance between copepods, corals, and bacteria without other groups with known homologues of the transporter makes the gene’s origin in copepods elusive. Before, it has been noted that “BCCT homologs were found only in a few animals hosting microalgal endosymbionts or close photosynthetic relatives” (Ngugi et al. 2020). If there are similar explanations for copepodids, remains to be seen. Due to the long evolutionary distance, the actual function of the protein must still be validated.

In adult *C. elongatus* a BHMT homologue was upregulated in low salinity. This encodes a betaine–homocysteine S-methyltransferase, which transfers a methyl group from glycine betaine to homocysteine, yielding dimethylglycine and methionine (Pajares and Pérez-Sala 2006). Using this enzyme, the lice could get rid of the unneeded osmolyte glycine betaine and adjust their internal osmolarity in low salinity. In rat hepatoma cells the mRNA expression of BHMT was found to be osmosensitive (Schäfer et al. 2007). When the cells were incubated in hypoosmotic medium, the expression of this gene was increased, in hyperosmotic medium, its expression was reduced. It has been suggested that adjustments of betaine removal could be involved in cell volume regulation (Hoffmann et al. 2009). In a coral species, BHMT is upregulated under hyposaline stress (Aguilar et al. 2019) and a similar observation was done in Chinese mitten crab (Shen et al. 2023). The concurrent downregulation of the BCCT transporter and the upregulation of BHMT could collaboratively contribute to the adjustment of the internal osmolarity in these animals.

### Ion transport

DEGs involved in ion transport include an epithelial sodium channel (ENaC) and a CLIC-like chloride channel. Given that sodium- and chloride-ions constitute NaCl, the most abundant salt in SW, it is anticipated that alterations in salinity would impact the transport of these ions within organisms. The ENaC-annotated gene was found to be downregulated in adults in BW. Interestingly its orthologue in *L. salmonis* (EMLSAG00000006377) was also downregulated in attached females in freshwater and BW (Borchel et al. 2021). Downregulation of this gene might thereby be a shared reaction pattern within the Caligidae. ENaCs are mainly characterized in mammals, and are known for their important role in sodium uptake in the kidney, and thereby regulating blood pressure (Pitzer et al. 2020). However, the exact classification of the *C. elongatus* gene is ambiguous. It has been stated that there are many members of the ENaC superfamily within invertebrates, but that these not necessarily are direct orthologues of the well-characterized mammalian ENaCs (Hanukoglu and Hanukoglu 2016).

In contrast to the sodium channel, a CLIK-like chloride channel was upregulated in BW in both copepodids and adults. In the pacific oyster the chloride transport of a CLIK channel was involved in the immune system (Zhang et al. 2020). Our results indicate that there might also be a role in osmoregulation or at least a function influenced by the osmotic state. In general, there seems to be a shift in membrane transport under low salinity as demonstrated by the enrichment of the GO-term “transmembrane transport” in downregulated genes in copepodids.

### Differences between active and moribund animals

At salinities between 21.4 ppt and 23.5 ppt, some of the adult female *C. elongatus* animals were classified as moribund, a state where they were not actively swimming. However, they were still alive as determined by observation of microscopic movements of the appendages and intestine. Concurrently other lice were still actively swimming or attached to the walls of their hatching well without showing any visible signs of distress. To investigate this observation further several of these samples were analysed by RNA-Seq to compare gene expression between active and moribund adult female lice in BW. Genes that are regulated in BW in comparison to SW in active animals, but not in moribund animals, may play an important part in successful osmoregulation in *C. elongatus*.

There were no differences in the concentration or purity of the RNA extracted between moribund and active animals, nor in the quality of samples or in the obtained reads in RNA-Seq. Apparently, the moribund state of the animals did not influence the integrity of the collected RNA.

Fifteen upregulated genes in BW were also significantly differentially expressed between active and moribund animals. This result from the RNA-Seq experiment was confirmed in a second independent experiment where four genes were analysed by qPCR showing the same differential regulation. These genes might play a key role in how the animals handle low salinities. A potential explanation for the differences might be that the moribund animals were unable to induce gene expression due to metabolic shut down. However, the upregulation of SIAH1 in moribund and active animals shows that also the moribund animals had been able to induce the gene expression of at least some genes. Therefore, it is still unclear, if the moribund state prevents the regulation of gene expression, or if the lack of gene expression adjustments (due to unknown reasons) leads to the moribund state. Nevertheless, the genes that were not upregulated in moribund, but in active animals, appear to be important for osmoregulation. Among these genes were genes encoding enzymes involved in proline synthesis (P5CR) and the glucose transporter, which was regulated by salinity in adults and copepodids, highlighting its importance. As mentioned, genes involved in proline synthesis are upregulated in low reduced salinity also in the salmon louse *L. salmonis* (Borchel et al. 2021) and in* T. californicus* (DeBiasse et al. 2018). That moribund animals of *C. elongatus* do not do this might suggest that an increase in proline synthesis might be necessary to successfully adapt to low salinities. However, further studies are required to understand osmoregulation taking place in marine parasitic copepods and how they handle variation in environmental salinity.

## Conclusions

*C.* *elongatus* displays a rather narrow salinity tolerance in adult females. Salinities under 24 ppt can render some animals moribund (24 h exposure), while salinities under 20 ppt kill the animals in less than seven hours. The planktonic copepodid stage is less sensitive: Few animals die at 15 ppt, but all animals were dead after 24 h at 10 ppt.

The molecular reactions of *C. elongatus* span regulation of proline synthesis, energy metabolism and cellular transport. Genes that were regulated in BW (23.5 ppt) in active animals, but not in moribund animals, like a glucose transporter, might be of special interest to further understand osmoregulation. Additionally, genes regulated in low salinity in both *C. elongatus* and the salmon louse (*L. salmonis),* like P5CR and the glycine betaine transporter, might have an evolutionary conserved function in marine copepod osmoregulation. The generation of the first transcriptome of *C. elongatus* will be of high value for future molecular studies on this species.

## Supplementary Information

Below is the link to the electronic supplementary material.

Supplementary file1 Table S1: Primers used for qPCR and their efficiencies (XLSX 14 KB)

Supplementary file2 File S2: MultiQC results (HTML 1572 KB)

Supplementary file3 File S3: Draft transcriptome (FASTA 163616 KB)

Supplementary file4 Table S4: Annotation of the draft transcriptome (XLSX 104703 KB)

Supplementary file5 Table S5: Results from differential expression analysis (XLSX 18832 KB)

## Data Availability

During the preparation of this work the authors used Microsoft Copilot in order to improve the language quality of the manuscript. After using this tool, the authors reviewed and edited the content as needed and take full responsibility for the content of the publication.
